# Incidence of paediatric multiple sclerosis and other acquired demyelinating syndromes: 10-year follow-up surveillance study

**DOI:** 10.1111/dmcn.15098

**Published:** 2021-10-24

**Authors:** Omar Abdel-Mannan, Michael Absoud, Christina Benetou, Helga Hickson, Cheryl Hemingway, Ming Lim, Sukhvir Wright, Yael Hacohen, Evangeline Wassmer, The UK Childhood Neuroinflammatory Disorders Network

**Affiliations:** 1 Queen Square MS Centre, UCL Queen Square Institute of Neurology, Faculty of Brain Sciences, University College London, London; 2 Department of Neurology, Great Ormond Street Hospital for Children, London; 3 Children’s Neurosciences, Evelina London Children’s Hospital, Guy’s and St Thomas’ NHS Foundation Trust, King’s Health Partners Academic Health Science Centre, London; 4 Department of Neurology, Birmingham Children’s Hospital, Birmingham; 5 Great Ormond Street Institute of Child Health, University College London, London; 6 Institute of Health and Neurodevelopment, College of Health and Life Sciences, Aston University, Birmingham, UK

## Abstract

**Aim:**

To describe a 10-year follow-up of children (<16y) with acquired demyelinating syndromes (ADS) from a UK-wide prospective surveillance study.

**Method:**

Diagnoses were retrieved from the patients’ records via the patients’ paediatric or adult neurologist using a questionnaire. Demyelinating phenotypes at follow-up were classified by an expert review panel.

**Results:**

Twenty-four out of 125 (19.2%) children (64 males, 61 females; median age 10y, range 1y 4mo–15y 11mo), identified in the original study, were diagnosed with multiple sclerosis (incidence of 2.04/million children/year); 23 of 24 fulfilled 2017 McDonald criteria at onset. Aquaporin-4-antibody neuromyelitis optica spectrum disorders were diagnosed in three (2.4%, 0.26/million children/year), and relapsing myelin oligodendrocyte glycoprotein antibody-associated disease in five (4%, 0.43/million children/year). Three out of 125 seronegative patients relapsed and 85 of 125 (68%) remained monophasic over 10 years. Five of 125 patients (4%) originally diagnosed with ADS were reclassified during follow-up: three children diagnosed initially with acute disseminated encephalomyelitis were subsequently diagnosed with acute necrotising encephalopathy (RAN-binding protein 2 mutation), primary haemophagocytic lymphohistiocytosis (*Munc 13-4* gene inversion), and anti-*N*-methyl-D-aspartate receptor encephalitis. One child initially diagnosed with optic neuritis was later diagnosed with vitamin B12 deficiency, and one presenting with transverse myelitis was subsequently diagnosed with Sjögren syndrome.

**Interpretation:**

The majority of ADS presentations in children are monophasic, even at 10-year follow-up. Given the implications for treatment strategies, multiple sclerosis and central nervous system autoantibody mimics warrant extensive investigation.

Acquired demyelinating syndromes (ADS) represent acute neurological illnesses characterized by deficits persisting for at least 24 hours and involving the optic nerve, brain, or spinal cord, associated with regional areas of increased signal on T2-weighted images.^1^ ADS may occur as a monophasic illness, such as optic neuritis, transverse myelitis, acute disseminated encephalomyelitis (ADEM), with an overall good prognosis,^2^ or as a chronic and relapsing condition, such as multiple sclerosis and neuromyelitis optica spectrum disorder (NMOSD), resulting in progressive disabilities.^3^

Diagnosis of multiple sclerosis, the most common relapsing form of ADS in both children and adults, requires evidence of inflammatory activity in more than one central nervous system (CNS) location (dissemination in space) in addition to recurrent disease over time (dissemination in time). The revised 2017 McDonald criteria allowed for intrathecal oligoclonal bands to substitute for dissemination in time, the inclusion of symptomatic lesions as evidence of dissemination in space/dissemination in time, and the inclusion of cortical grey matter lesions in dissemination in space.^4^ These criteria also highlighted the importance of excluding alternative diagnoses, and have a high specificity and sensitivity in the paediatric population, including children younger than 12 years not presenting with ADEM.^5,6^

Many patients with NMOSD also have frequent relapses. Aquaporin-4 antibodies (AQP4-Ab) have been identified in NMOSD, which has led to a more rapid initiation of treatment.^7^ In approximately 40% of children with ADS, myelin oligodendrocyte glycoprotein antibodies (MOG-Ab) have been reported.^8^ Although largely associated with monophasic illness, MOG-Ab have also been associated with relapses and detected in patients with multiphasic disseminated encephalomyelitis,^9^ recurrent idiopathic optic neuritis,^10^ and ADEM optic neuritis (ADEMON), which can be followed by recurrent or monophasic optic neuritis.^11^ Identification and distinction of the different subtypes of ADS, especially at first presentation, has important implications for treatment and prognosis,^12^ with accurate diagnosis and management of inflammation being key to improving patient outcomes.^4^

In a UK-wide prospective surveillance study of children under the age of 16 years (September 2009–September 2010), the incidence of childhood CNS inflammatory demyelination was calculated as 9.83/million/year.^13^ Here, we conducted 10-year follow-up evaluations of the same cohort to ascertain the incidence of multiple sclerosis and other relapsing demyelinating syndromes.

## Method

As detailed previously,^13^ children under the age of 16 years, with a first episode of ADS, evaluated with brain and/or spine magnetic resonance imaging (MRI), were ascertained from a prospective national UK surveillance study (2009–2010) using two well-established surveillance units: the British Paediatric Surveillance Unit and the British Ophthalmological Surveillance Unit. Serum MOG-Ab and AQP4-Ab were not routinely tested. Serum AQP4-Ab was only requested when NMOSD was suspected clinically and MOG-Ab testing was additionally performed on these samples, as previously reported (*n*=49).^8^ Acute samples were taken within 3 months of clinical presentation^8^ and samples were stored at –80°C. Additional MOG-Ab testing was requested once the test became clinically available in 2014 (*n*=27).

Demyelinating phenotypes were classified by an expert review panel (EW, ML, SW, YH) at onset^13^ and on final follow-up on the basis of International Paediatric Multiple Sclerosis Study Group criteria,^1^ the revised 2017 McDonald criteria for the diagnosis of multiple sclerosis,^4^ and International Consensus Diagnostic Criteria for NMOSD.^7^ Clinical data, paraclinical data, and final diagnosis at 10 years were retrieved from the patients’ medical records via the patients’ primary paediatric or adult neurologist, using a questionnaire.

Ethical approval for the surveillance study was given by the UK Multicentre Research Ethics Committee (09/H1202/92).

### Statistical analysis

Descriptive statistics were used to summarize the key components of the data set. Non-parametric statistical tests (Kruskal–Wallis tests) were used for continuous distributions, as appropriate, given the lack of normality and χ^2^ or Fisher’s exact tests were used for nominal data. Estimates of national incidence with confidence intervals (Byar’s approximation of the exact Poisson^14^) for the 13-month study were annualized using mid-2010 UK and 2010 Republic of Ireland population estimates.^15^ Analyses were performed using GraphPad Prism 8 (GraphPad Software, San Diego, CA, USA).

## Results

**Figure 1:**
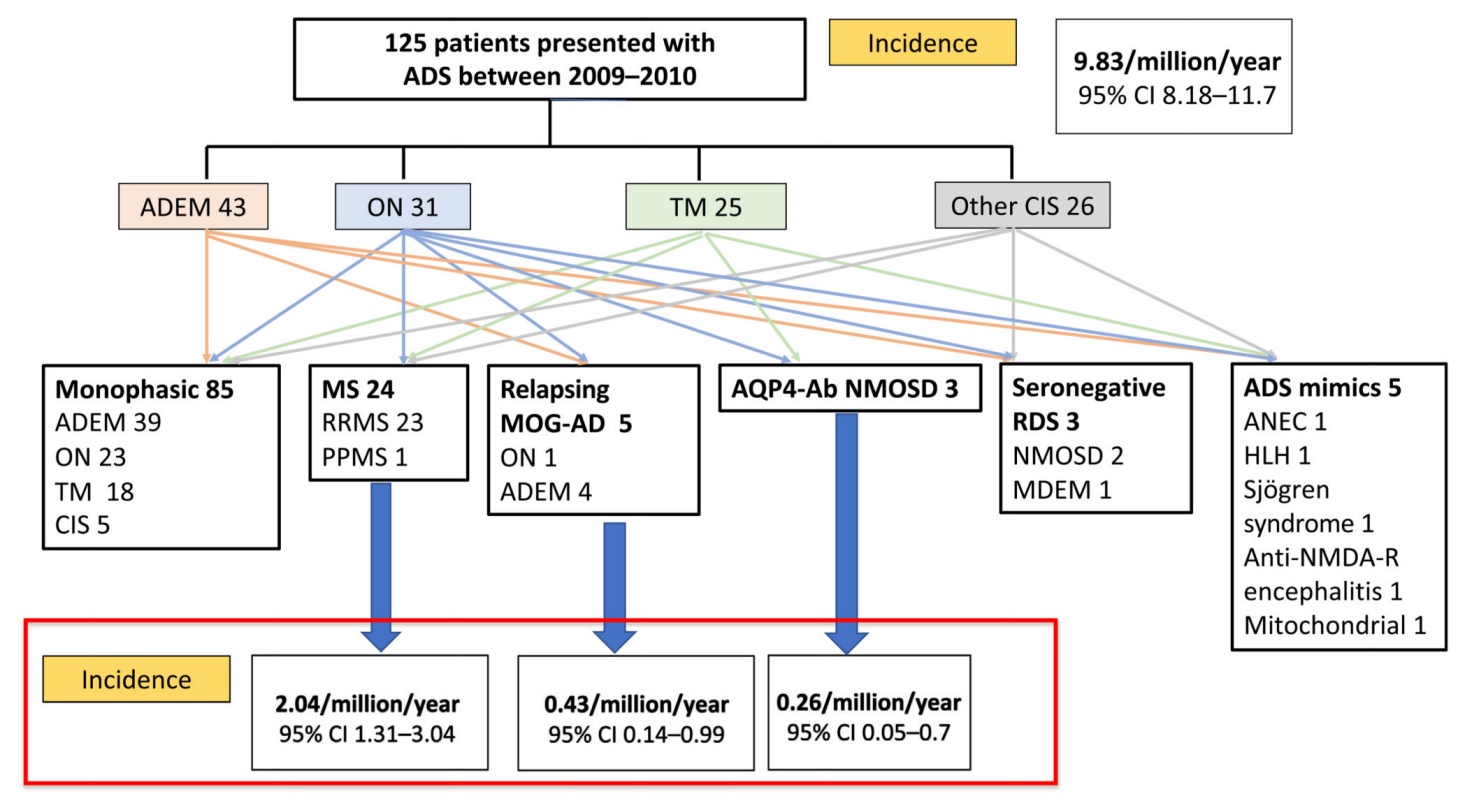
A total of 125 children were included in the original cohort. Initial presentations were acute disseminated encephalomyelitis (ADEM) in 43 (34.4%), optic neuritis (ON) in 31 (24.8%), transverse myelitis (TM) in 25 (20%), and other clinically isolated syndrome (CIS) presentations in 26 patients (20.1%). At 10-year follow-up, 85 (68%) of the 125 children included had a monophasic acquired demyelinating syndrome. Thirty-five children (28%) had a relapsing demyelinating syndrome (RDS); 24 had a final diagnosis of multiple sclerosis (MS), four had relapsing myelin oligodendrocyte glycoprotein antibody-associated disease (MOG-AD), three had aquaporin-4 antibody (AQP4-Ab) positive neuromyelitis optica spectrum disorder (NMOSD), and three had seronegative RDS. Incidence was calculated for MS, relapsing MOG-AD, and AQP4-Ab NMOSD and is shown below the relevant diagnoses. ANEC, acute necrotising encephalopathy; HLH, haemophagocytic lymphohistiocytosis; MDEM, multiphasic disseminated encephalomyelitis; RRMS, relapsing remitting multiple sclerosis; PPMS, primary progressive multiple sclerosis; NMDA-R, *N*-methyl-D-aspartate receptor.

A total of 125 children (64 males, 61 females; median age 10y, range 1y 4mo–15y 11mo), were included in the original cohort; follow-up data on diagnosis at 10 years were collected. Paediatric and adult neurologists responded with completed questionnaires for 113 of 125 patients (90%). Of the 12 patients who did not have 10-year follow-up data (optic neuritis *n*=6, transverse myelitis *n*=3, ADEM *n*=3), all remained monophasic at 3 years. Eighty-five (68%) of the 125 children included had a monophasic ADS, of which 39 (45.8%) presented with ADEM, 23 (27.1%) with optic neuritis, 18 (21.2%) with transverse myelitis (four with short transverse myelitis and 14 with longitudinally extensive transverse myelitis), and five (5.9%) with other clinically isolated syndrome presentations (Fig. 1).

Thirty-five children (28%) had relapsing demyelinating syndromes. Twenty-four children (19.2%) had a final diagnosis of multiple sclerosis, including 23 with relapsing remitting multiple sclerosis, and one had a primary progressive phenotype. Therefore, the incidence of multiple sclerosis in children under the age of 16 years in the UK and Republic of Ireland was calculated as 2.04/million/year (95% confidence interval [CI] 1.31–3.04). Of these, four of 24 presented under the age of 12 years, with a UK incidence of 0.34/million children/year (95% CI 0.09–0.87). When retrospectively applied, the revised 2017 McDonald’s diagnostic criteria for diagnosis of multiple sclerosis could be made at presentation in 23 of 24 patients. The remaining patient met the dissemination in space criterion at presentation (i.e. did not have a contrasted scan or a lumbar puncture) and had a clinical relapse within 1 year of disease onset.

Only 76 of 125 (60.8%) patients had MOG-Ab and AQP4-Ab tested, of which 20 of 76 (26.3%) were positive for MOG-Ab and three of 76 (3.9%) for AQP4-Ab. All 24 patients with a diagnosis of multiple sclerosis, including the four patients with disease onset under the age of 12 years, were negative for both autoantibodies. Antibodies were tested in 41 of 85 patients with a monophasic disease compared to 35 of 35 patients with a relapsing disease. Of the 41 patients with a monophasic disease who had antibody testing, 37 were tested at onset, and a further four were tested at follow-up. Only 14 of 43 patients presenting with ADEM had antibody testing at onset and a further three at follow-up. Nineteen of 35 patients with a relapsing disease course who had antibody testing were tested at onset and a further 16 were tested at follow-up. Five of 20 of the MOG-Ab positive patients had a relapsing disease course. Therefore, the incidence of relapsing MOG-Ab-associated disease in children was calculated as 0.43/million children/year (95% CI 0.14–0.99) and that of AQP4-Ab NMOSD as 0.26/million children/year (95% CI 0.05–0.7).

**Figure 2:**
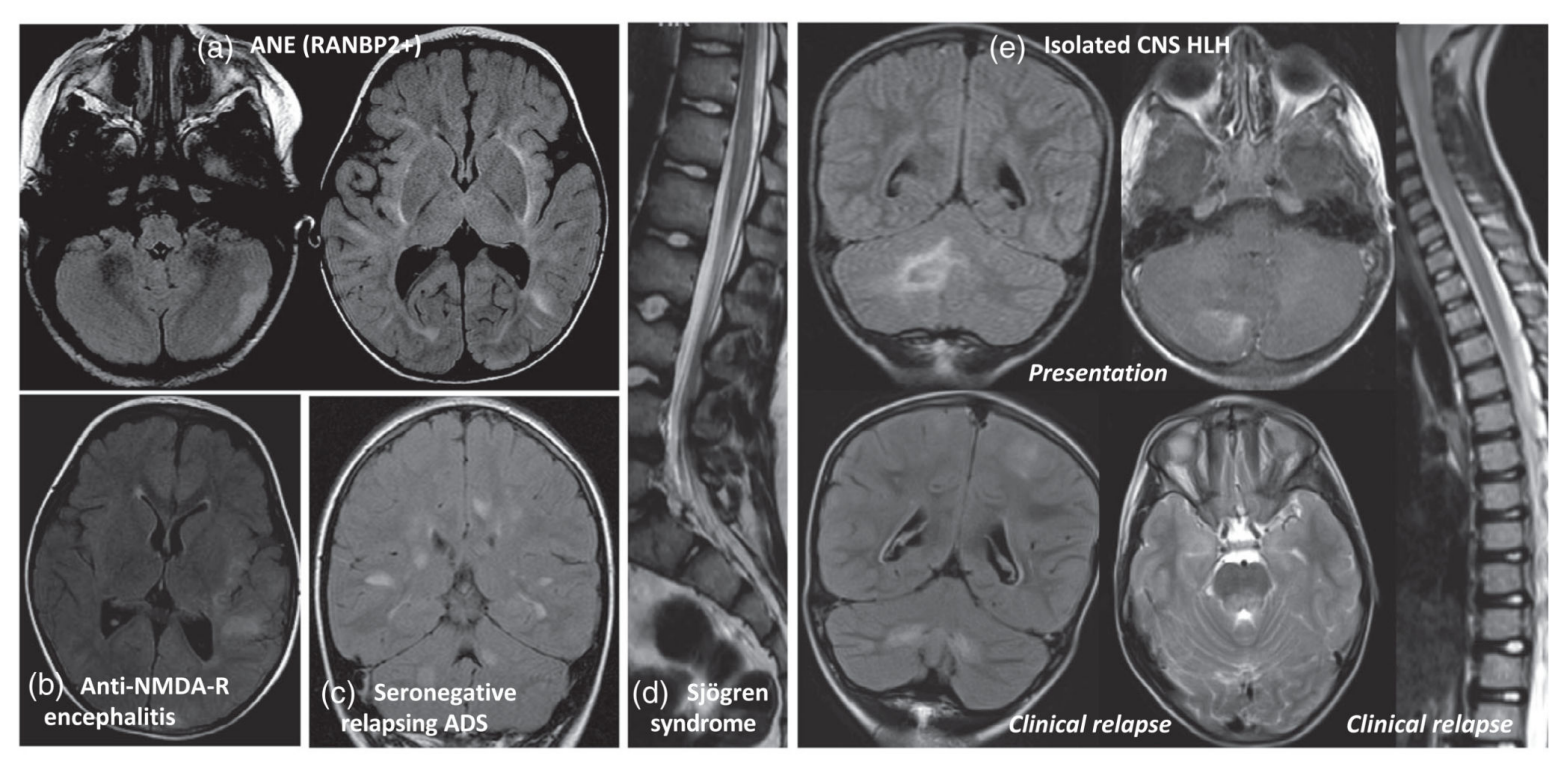
Five cases with alternative diagnoses at 10-year follow-up. (a) A female patient presented at age 16 months with encephalopathy, generalized seizures, abnormal eye movements, and hypotonia. The sister also presented previously with acute disseminated encephalomyelitis at a similar age. Axial T2-weighted fluid attenuated inversion recover (FLAIR) brain magnetic resonance imaging (MRI) showed abnormal hyperintensity of the left cerebellar grey matter and fairly symmetrical hyperintensity of the cerebral white matter, including the external capsules. Genetic screening confirmed *RANBP2* mutation in both siblings. (b) A female patient presented at age 2 years with severe encephalopathy, seizures, and a complex movement disorder. The patient was noted to have a right-sided hemiparesis on clinical examination. The patient was positive for serum anti-*N*-methyl-D-aspartate receptor (NMDA-R) antibodies, and negative for myelin oligodendrocyte glycoprotein-antibodies. Axial T2-weighted FLAIR brain MRI showed an asymmetric distribution of multiple hyperintense grey and white matter lesions, with a notable grey matter predominance. (c) A female patient presented at 14 years of age with encephalopathy, bilateral weakness, sensory loss with cerebrospinal fluid protein elevation (2.6g/L), and positive oligoclonal bands. Axial T2-weighted brain MRI showed an extensive bilateral, asymmetrical, patchy parenchymal signal abnormality involving the deep white matter, brainstem, internal capsules, and cerebellar peduncles. The patient also had longitudinally extensive transverse myelitis (LETM) from C1 to T4 (not shown here). The patient went on to have two further relapses with a similar presentation within the first year. (d) A female patient presented at 12 years of age with bilateral weakness of upper and lower limbs in addition to sphincter dysfunction. Sagittal T2-weighted MRI of the spinal cord showed a lesion in the conus medullaris. (e) A male patient presented at 8 years of age with left convergent squint, ataxia, seizures in addition to lung infiltrates, cycling cytopenia, and hepatosplenomegaly. Coronal T2-weighted FLAIR and contrast enhanced T1-weighted brain MRI at presentation showed a heterogeneously enhancing lesion in the right cerebellar hemisphere bearing some localized oedema and leptomeningeal enhancement. Follow-up imaging on relapse showed symmetrical hyperintense lesions on T2-weighted images in the cerebellum and dorsal pons, symmetrical lesions in the cerebral hemispheres as well as an LETM. The patient underwent a bone marrow transplant with an unsuccessful central nervous system response with clinical and neurological evidence of progression that led to death.

Five patients originally diagnosed with ADS had an alternative diagnosis at 10-year follow-up. Three patients originally diagnosed with ADEM were found to have the following three final diagnoses: acute necrotising encephalopathy with a confirmed mutation in *RANBP2*, primary haemophagocytic lymphohistiocytosis with an inversion of the *Munc 13-4* gene, and anti-*N*-methyl-D-aspartate receptor encephalitis (with white matter involvement on neuroimaging) (Fig. 2). One patient initially diagnosed with optic neuritis did not respond to immunotherapy and was diagnosed with vitamin B_12_ deficiency (with a concurrent mitochondrial ND5 variant). Finally, one patient who presented with transverse myelitis was subsequently diagnosed with Sjögren syndrome.

Table 1 includes clinical and paraclinical information for this cohort. Children in the monophasic ADS group were younger than the multiple sclerosis cohort (median age 8y 8mo vs 13y 11mo, *p*<0.001) and were more likely to present with ADEM; none of the children presenting with ADEM were subsequently diagnosed with multiple sclerosis. Abnormalities in brain MRI at presentation were seen in 23 of 24 patients with multiple sclerosis compared to 50 of 83 patients in the monophasic group (*p*<0.001). Intrathecal oligoclonal bands were reported in 24 of 24 of the multiple sclerosis group compared to only four of 53 (7.5%) of the monophasic ADS group (*p*<0.001).

Three children (2.4%) died during follow-up; one patient during acute presentation of ADEM from acute fulminant inflammation inducing cerebral oedema, one with AQP4-Ab NMOSD 10 years after initial presentation during relapse following a hyperkalaemic cardiac arrest, and the patient with primary haemophagocytic lymphohistiocytosis died after an unsuccessful bone marrow transplant with further CNS relapses.

## Discussion

In this 10-year follow-up of a UK population active surveillance study of children with ADS, we have shown that the majority of the children had a monophasic course. The key observation is that almost all the children with multiple sclerosis (95.8%) met the 2017 revised McDonald diagnostic criteria at presentation. Of note, oligoclonal band analysis and contrasted scans were not performed for the one patient who did not fulfil the criteria at onset. At the time of the initial study, 2006 McDonald criteria were being used for multiple sclerosis diagnosis, which have subsequently been superseded by both 2010 and 2017 McDonald criteria, with improved sensitivities for both adults^16^ and children.^5,6,17^ In fact, 10 of 24 cases fulfilled 2010 McDonald criteria at the time of the study. The annual incidence of multiple sclerosis in children younger than 16 years (2.04/million children) in our cohort is similar to that reported in a number of other studies internationally, ranging from 0.13 to 2.85/100 000 children/year.^18–20^

Since the initial surveillance period in 2009 and 2010 there has been a paradigm shift in the diagnosis and management of relapsing demyelinating syndromes of childhood, given the discovery of CNS autoantibodies, with both AQP4-Ab NMOSD and MOG-Ab-associated disease being increasingly recognized.^12^ The low incidence of MOG-Ab positivity and the small proportion of relapsing MOG-Ab-associated disease in this cohort is likely to be due to the fact that MOG-Ab testing only became clinically available from 2014. In fact, despite the prevalence of MOG-Ab positivity being reported highest across ADS phenotypes,^21^ only 17 of 43 children presenting with ADEM had MOG-Ab tested, which is likely to explain the low MOG-Ab positivity reported here. Furthermore, patients who had antibodies tested in 2009 and 2010 were more likely to have a relapsing disease, and within the monophasic group it is possible that by the time of testing they had already become seronegative.^22^ Recent data suggest that up to one-third of children with ADS have MOG-Ab positivity^23^ and the proportion of MOG-Ab positive patients with a relapsing disease course in this cohort is similar to that reported in other prospective cohorts.^22,24^

Our reported paediatric incidence of AQP4-Ab NMOSD (0.26/million children/year) is similar to that reported in paediatric studies worldwide (ranging from 0.01–0.06/100 000/year);^25^ however, data remain scarce in this group. NMOSD has global variations in both prevalence and incidence among different geographic areas and ethnicities. In two UK studies in small areas of England and Wales,^26,27^ the prevalence of NMO/NMOSD was calculated as 19.6/million (95% CI 1.22–2.97), with 21% of the reported prevalent cases under 20 years of age, resulting in a higher prevalence in the age group from 0 to 19 years.

Of note, five children who were initially reported in the ADS cohort were found to have alternative inflammatory aetiologies, with important treatment implications. Although traditionally patients with monogenetic disorders have been thought to be younger, to have underlying developmental delay, symmetrical MRI abnormalities, and lack of response to immunosuppression, we now recognize an increasing number of conditions mimicking ADS. Notably, the patient with primary haemophagocytic lymphohistiocytosis had a relapsing disease course, good response to steroids, and fulfilled both diagnostic criteria for multiple sclerosis and NMOSD. In a study of 322 patients with ADS from the Canadian Pediatric Demyelinating Disease Network, 20 children (6%) were ultimately diagnosed with alternative diagnoses.^28^ In contrast to our report, the most commonly reported diagnosis in 11 of those 20 patients was vascular disorders (primary or secondary CNS vasculitis, vasculopathy, stroke, or migraine). Malignant brain tumours are also an important, if rare, differential to bear in mind.^28^

The study is limited, as with other epidemiological studies, by the potential underreporting of cases; however, this was largely addressed by using clear consensus case definitions and multiple sources of case ascertainment. As seen in our study, loss to follow-up can be an issue with epidemiological studies due to patient migration; however, the UK national healthcare system has allowed us to identify the majority of patients (90% from clinician responses). In addition, it is unlikely that patients labelled initially as monophasic ADS would have had further relapses and not been referred to clinicians within the NHS England Highly Specialised Service for Paediatric Multiple Sclerosis. This service includes children with multiple sclerosis and other recurrent demyelinating syndromes. Another limitation was the lack of systematic antibody testing at onset and long-term follow-up data on disability (e.g. using the Expanded Disability Status Scale) and other parameters. Nevertheless, our study clearly demonstrates that (1) the majority of ADS presentations in children are monophasic, and (2) the diagnosis of multiple sclerosis can be made at onset in the majority of cases when cerebrospinal fluid and/or contrasted scans are available. This is relevant when counselling young people and their families at presentation. Understanding the actual ‘real-world’ burden of individual demyelinating conditions by geographic location, age, sex, and ethnicity will facilitate more accurate diagnostics, effective treatment and advice, resource allocation, and service development. Given the implications for treatment strategies, extensive investigations are crucial in paediatric ADS presentations to ensure earlier diagnosis of multiple sclerosis and antibody-mediated demyelinating disorders.

## Supplementary Material

The following additional material may be found online: **Appendix S1:** Members of the UK Childhood Neuroinflammatory Disorders Network.

Appendix S1

## Figures and Tables

**Table 1:** Clinical and paraclinical features of monophasic ADS, multiple sclerosis, and all patients

	All patients (n=125)	Monophasic ADS (n=85)	Multiple sclerosis (n=24)	*p* (monophasic ADS vs multiple sclerosis
Age at presentation, y:mo, median (IQR)	10:4 (6:5-13:11)	8:8 (5:11-12:2)	13:11 (12:10-14:8)	<0.001
Sex, male:female (ratio)	64:61 (1.05:1)	43:42 (1.02:1)	9:15 (1:1.7)	0.35
Ethnicity, white:other (ratio)	102:23 (4.4:1)	71:14 (5.1:1)	16:8 (2:1)	0.06
Presentation, *n* (%)				
ADEM	43 (34.4)	39 (45.9)	0 (0)	<0.001
Transverse myelitis	25 (20)	18 (21.2)	4 (16.7)	0.57
Optic neuritis	31 (24.8)	23 (27.1)	7 (29.2)	0.93
CIS – other	26 (20.1)	5 (5.9)	13 (54.2)	<0.001
CSF OCB, *n* (%)	24/80 (30)	4/53 (7.5)	17/17 (100)	<0.001
Abnormal brain MRI at onset, *n* (%)	83/121 (68.6)	50/83 (60.2)	23/24 (95.8)	<0.001
Abnormal spine MRI at onset, *n* (%)	29/58 (50)	26/41 (63.4)	8/11 (72.7)	0.63

ADS, acquired demyelinating syndromes; IQR, interquartile range; ADEM, acute disseminated encephalomyelitis; CIS, clinically isolated syndrome; CSF, cerebrospinal fluid; OCB, oligoclonal bands; MRI, magnetic resonance imaging.

## Data Availability

Data are available upon reasonable request. The deidentified participant data are available from the corresponding author. Both centre and department have to give the permission to reuse the database.

## What this paper adds

- The majority of paediatric acquired demyelinating syndrome (ADS) presentations are monophasic, even at 10-year follow-up.
- UK paediatric multiple sclerosis incidence is 2.04/million children/year.
- Myelin oligodendrocyte glycoprotein antibody-associated disease and Aquaporin-4-antibody neuromyelitis optica spectrum disorder incidence was similar to previous cohorts.
- Almost all patients (95.8%) met the 2017 McDonald criteria for multiple sclerosis at presentation.
- Five children initially reported in the ADS cohort had alternative inflammatory aetiologies.

## Abbreviations

ADEM: Acute disseminated encephalomyelitis
ADS: Acquired demyelinating syndromes
AQP4-Ab: Aquaporin-4 antibody
MOG-Ab: Myelin oligodendrocyte glycoprotein antibody
NMOSD: Neuromyelitis optica spectrum disorder
