# Supplementary material for: Incidence of paediatric multiple sclerosis and other acquired demyelinating syndromes: 10-year follow-up surveillance study
Source: Dev Med Child Neurol. Author manuscript; Available in PMC 2025 Sep 4. (PMC7618074; doi:10.1111/dmcn.15098)
Supplement: Appendix S1 [file EMS208261-supplement-Appendix_S1.docx]

**Appendix S1**

Members of the UK Childhood Inflammatory Demyelination Network

Michael Taylor, Department of Neurology, Alder Hey Children’s NHS Foundation Trust, Liverpool; Manali Chitre, Department of Paediatric Neurology, Addenbrooke’s Hospital, Cambridge; W K Chong, Department of Neurology, Alder Hey Children’s NHS Foundation Trust, Liverpool; Carole Cummins, Institute of Applied Health Research, University of Birmingham, Birmingham; Christian De Goede, Paediatric Neurology, Royal Preston Hospital, Lancashire; Katharine Forrest, Paediatric Neurology, Royal Hospital for Children, Glasgow; Rob Forsyth, Paediatric Neurology, Newcastle upon Tyne Hospitals NHS Foundation Trust, Newcastle; Philip E Jardine, Paediatric Neurology, University Hospitals Bristol and Weston NHS Foundation Trust, Bristol; Rachel Kneen, Department of Neurology, Alder Hey Children’s NHS Foundation Trust, Liverpool and Institute of Infection and Global Health, University of Liverpool; Marcus Likeman, Neuroradiology, University Hospitals Bristol and Weston NHS Foundation Trust, Bristol, UK. Bryan Lynch, Paediatric Neurology, Temple Street Children's University Hospital, Dublin, Republic of Ireland. Santosh Mordekar, Department of Paediatric Neurology, Sheffield Children's Hospital, Sheffield, UK. Ken Nischal, Pediatric Neurology, UPMC Children's Hospital of Pittsburgh, Pittsburgh, USA. Michael G Pike, Department of Paediatric Neurology, John Radcliffe Hospital, Oxford; Sithara Ramdas, Department of Paediatric Neurology, John Radcliffe Hospital, Oxford; Dipak Ram^,^ Department of Neurology, Royal Manchester Children’s Hospital, Manchester; Naomi Sibtain, Neuroradiology, King’s College Hospital NHS Foundation Trust, London; Kayal Vijayakumar, Paediatric Neurology, University Hospitals Bristol and Weston NHS Foundation Trust, Bristol; Siobhan West, Department of Neurology, Royal Manchester Children’s Hospital, Manchester; William P Whitehouse, School of Medicine, University of Nottingham and Paediatric Neurology, Nottingham Children’s Hospital, Nottingham University Hospitals NHS Trust, Nottingham, UK.
